# Impediments to Meniscal Repair: Factors at Play Beyond Vascularity

**DOI:** 10.3389/fbioe.2022.843166

**Published:** 2022-03-01

**Authors:** Jay M. Patel

**Affiliations:** ^1^ Department of Orthopaedics, Emory University School of Medicine, Atlanta, GA, United States; ^2^ Atlanta VA Medical Center, Department of Veterans Affairs, Decatur, GA, United States

**Keywords:** meniscus, meniscus repair, mechanobiolgy, tissue engineering, vascularity

## Introduction

The menisci are semi-lunar wedge-shaped discs that are vital to load distribution, stability, and lubrication of the knee (Fox et al., 2012). Due to the variety of stresses placed on these tissues, they are often injured, both through trauma and degeneration. Due to the relative avascularity in the tissue (Henning et al., 1987), it mostly lacks the capacity to self-heal, necessitating surgical intervention, with nearly 500,000 arthroscopic meniscal procedures annually in the US alone (Kim et al., 2011). Meniscectomy, or removal of the torn tissue, remains a leading treatment modality (Abrams et al., 2013; DeFroda et al., 2020), as it provides symptomatic relief from catching and locking; however, it predisposes the joint to long-term degeneration due to increased stresses placed on the articular surfaces (McDermott and Amis, 2006; Wang et al., 2015). Meniscus replacement options, such as allografts and scaffolds (Rodeo, 2001; Steadman and Rodkey, 2005; Efe et al., 2012; Lee et al., 2012), certainly exist, but they are currently limited in their long-term efficacy due to lacking formation and/or maintenance of functional meniscus tissue. For this reason, meniscal suture repair to preserve the native tissue has become increasingly popular (Beaufils and Pujol, 2017; Momaya, 2019), yet these procedures are only performed at a fraction of the rate (10–15%) of meniscectomy.

**FIGURE 1:**
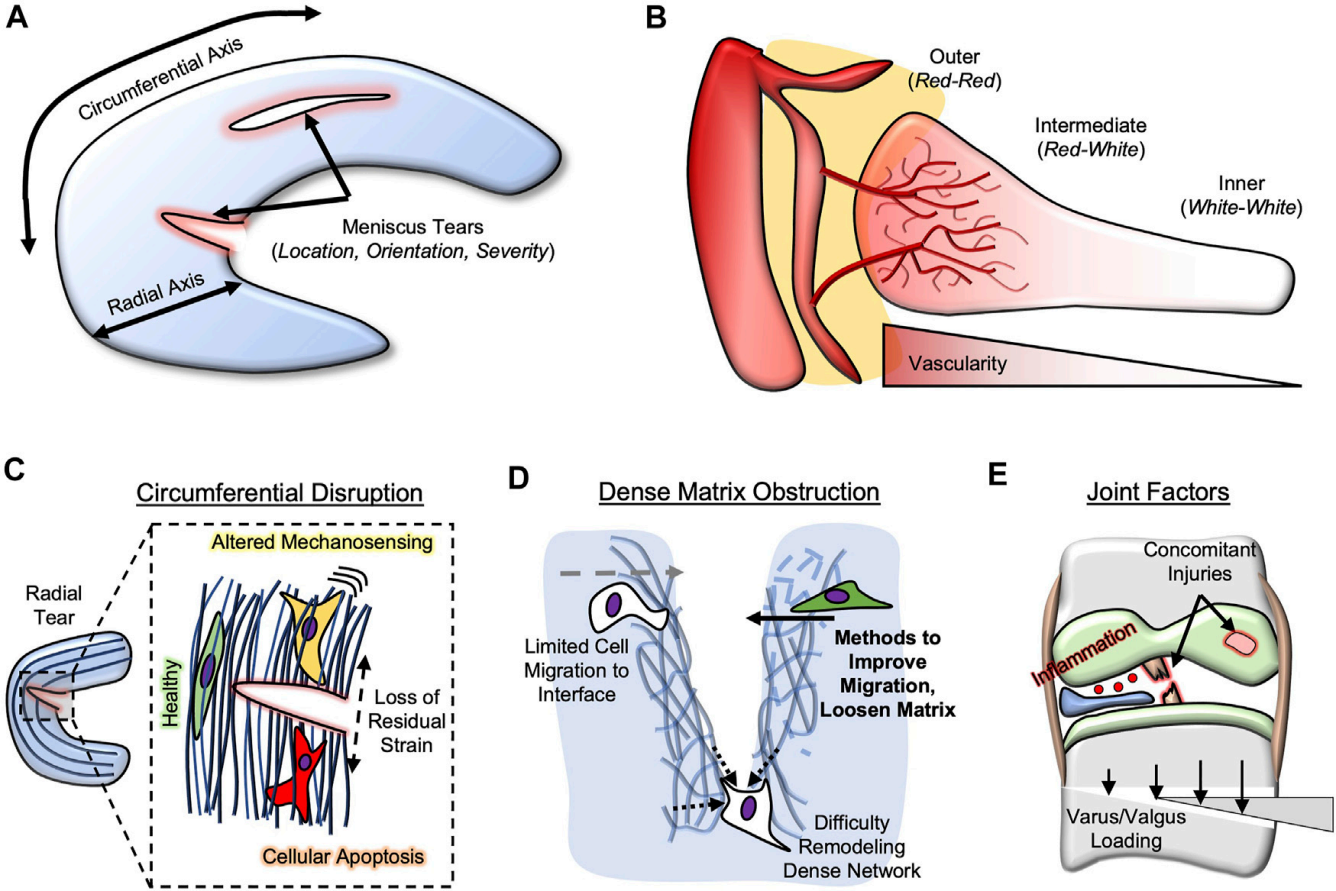
Meniscus Repair Impediments. **(A)** Meniscus tears are often classified based on location, orientation, and severity. Location is usually classified by radial axis (inner, intermediate, outer) and circumferential axis (anterior, body, posterior). **(B)** Meniscus vascularity penetrates only partially into the meniscal body. **(C)** Circumferential network disruption (radial tears) lead to loss of residual strain, resulting in altered mechano-sensing and potentially cellular apoptosis. **(D)** Dense extracellular matrix can obstruct repair by limiting cell migration and matrix remodeling. Methods (e.g., growth factors) can improve migration and loosen matrix. **(E)** Joint factors that influence meniscal repair include concomitant injuries (e.g., ACl, cartilage), inflammation, and varus/valgus loading.

The decision to perform meniscectomy versus suture repair is often predicated on the region, geometry, and severity of the tear (Figure 1A). Furthermore, there are often many patient-level and joint-level factors that influence a clinician’s decision-making process. For example, degraded meniscal tissue in older patients may be treated more conservatively to provide symptomatic relief as opposed to a younger patient with more acute tears, where the goal would be to preserve the meniscus and its function. Other factors such as comorbidities (e.g., cartilage wear, ligament status, alignment) may also factor into this decision. Even in a relatively healthy patient, there are many complex and challenging tear types, such as posterior root tears, that cannot simply be repaired. Here, we focus on acute tears within the body of the meniscus, where a surgeon often decides between meniscectomy and suture repair. In this subset, tears in the outer half of the meniscus, which is relatively more vascular (Henning et al., 1987), are mostly repaired, since the vascular supply is thought to provide enough nutrients to naturally bridge the tissue gap following suturing. However, since tears in the inner half (almost devoid of vascularity) lack access to this blood supply, the torn tissue is typically removed to alleviate symptoms (Henning et al., 1987; Mordecai, 2014), since suturing the torn edges may not result in eventual tissue bridging. This inner vs. outer dogma of meniscus repair has long governed mode of injury management; however, findings from the musculoskeletal research field may challenge this philosophy as the sole player. The purpose of this opinion article is to extend the impediments of meniscal repair beyond the traditional inner vs. outer paradigm, suggesting the role of other factors: disruption of the circumferential network, dense matrix as an obstruction to tissue joining, and other joint pathologies that may influence repair quality.

## The Inner Versus Outer Paradigm

The meniscus is often divided into zones along the radial axis, perpendicular to the circumferential network (Figure 1B). Often, the outer meniscus is deemed the red zone, as it contains vascular structures that penetrate, from the meniscosynovial junction, into the tissue. These vessels terminate in the middle third of the meniscus, deemed the red-white zone, leaving the inner third to half of the meniscus devoid of blood supply (termed the white zone). Studies comparing inner to outer meniscus healing rates are few in number (Cinque et al., 2019), perhaps since inner meniscus tears have not historically been repaired. Thus, the recent push to “save the meniscus” is complicated by the majority of tears occurring in either the red-white or white zones (Terzidis et al., 2006), limiting repair potential with suturing. Further exacerbating this issue is that the posterior horn of the meniscus appears to be most injured (Mansori et al., 2018; Jackson et al., 2019), yet exhibits the lowest vascular penetration (Crawford et al., 2020).

The role of vascularity in outer meniscal healing seems to rely on a wound healing response from blood supply, as well as the availability of stimulating growth factors, such as hypoxia inducible factor-1 (HIF-1a) and vascular endothelial growth factor (VEGF) (Lu et al., 2017). For this reason, a plethora of basic science researchers have attempted to augment inner meniscal repair using these vascular-derived factors. Meniscal “perforations”, or surgical holes punctured from the inner-zone meniscus tear outwards towards the periphery, have been attempted preclinically and clinically (Zhongnan et al., 1988; Cook and Fox, 2007), albeit with inconsistent improvement in outcomes. Platelet-rich plasma and bone marrow aspirate have both been widely utilized in conjunction with avascular meniscal tears (Griffin et al., 2015; Muckenhirn et al., 2017; Kaminski et al., 2018), further highlighting the propensity towards “recreating vascularity” to enhance healing of inner meniscal tears. Certainly, vascularity is a player in meniscal healing, but is it the only one? Findings from the musculoskeletal field suggest that there may be others involved, and these may need to be considered to advance the meniscal repair field.

## Disruption of Circumferential Collagen Network

As mentioned previously, the menisci are semi-lunar fibrocartilage tissues, and perhaps most important to their function is their array of circumferentially-aligned collagen fibers (Bullough et al., 1970; Fithian et al., 1990). This organization enables the tissue to distribute loads in the knee by generating circumferential hoop stresses (Lee et al., 2006). Meniscus tears occur in a variety of orientations relative to this network; vertical and horizontal tears are parallel to the circumferential arrangement, while radial tears are perpendicular. Thus, radial tears disrupt the aligned collagen network; disruption of similar networks in other aligned tissues, such as tendon and annulus fibrosus, have been shown to be especially problematic. For example, annulus fibrosus cells exhibit aberrant behavior, including fibrotic phenotypic changes (alpha-smooth muscle actin expression) and even apoptosis, following removal of residual strains (Bonnevie et al., 2019). Similarly, transection of tendons (e.g., rotator cuff (Osti et al., 2017)) perpendicular to the aligned axis leads to similar fibrotic and apoptotic behavior (Egerbacher et al., 2008; Maeda et al., 2011; Lundgreen et al., 2013; T. et al., 2020). Thus, it is expected that radial meniscus tears may cause similar cellular changes (Figure 1C), especially near the lesion site, altering their capacity for healing. Of interest is that there seems to be a trend towards more radial tears in the lateral meniscus (Terzidis et al., 2006), which also exhibits a greater number of white-zone tears than the medial meniscus, potentially implicating circumferential network disruption, and not avascularity alone, as a player in lower healing capacity of the inner meniscus. Differences in the medial vs. lateral meniscus could also be influential in a surgeon’s management; the lateral meniscus displaces more during loading (Bylski-Austrow et al., 1994) and less force is typically transmitted through the lateral compartment (Zhao et al., 2007), meaning that circumferential disruption could present a greater issue in the medial meniscus. Clinical systematic reviews, and perhaps preclinical animal studies (Bansal et al., 2020), that investigate the healing rates of inner vs. outer zone radial tears, and inner vertical vs. inner radial tears, would help to test this hypothesis.

## Dense Extracellular Matrix as an Obstructor to Healing

One of the greatest obstacles in the field of musculoskeletal repair is the re-integration of wound edges. Frequently thought of in the context of tissue engineering [scaffold-to-tissue integration; (Moffat et al., 2009)], healing of meniscal tears requires two edges of the meniscus to join back together. Suturing holds these edges together initially, but long-term bridging of this gap will require a combination of tissue deposition and remodeling along the interface. A plethora of researchers have attempted to improve meniscal repair healing with scaffolds [e.g., fibrin, collagen, electrospun polymers (Scotti et al., 2009; González-Fernández et al., 2016; Baek et al., 2019)] that are often supplemented with cells (meniscal fibrochondrocytes, marrow stromal cells) and factors (transforming growth factor-beta 3, connective tissue growth factor) (He et al., 2011; Cucchiarini et al., 2015; Sasaki et al., 2018), yet the overall shear strengths of this repair interface are typically orders of magnitude lower than the native meniscus, leaving it susceptible to re-tear. A potential obstructor in this repair interface is the dense nature of native meniscal tissue; the dense matrix limits cell migration to, and eventual healing at, the tear site, and it may lack the capacity to undergo active remodeling to integrate the two edges (Figure 1D).

Many techniques have been employed to improve meniscus cell migration to improve integrative repair. This is especially important in older patients, as both cell motility and proliferation decrease with age (Bartling et al., 2009; Qu et al., 2019), affecting repair potential and efficacy. For example, resident meniscus cells can be “activated” with electrical stimulation (Gunja et al., 2012; Yuan et al., 2014), growth factor delivery (e.g., platelet derived growth factor; (Qu et al., 2017)), or perhaps the addition of exogenous cells/biologics [e.g., endothelial cells (Yuan et al., 2015), platelets (Wong et al., 2017), hyaluronic acid (Murakami et al., 2019)]. A portion of the field is also studying a subpopulation of meniscal progenitor cells that further aid in the process of regeneration (Muhammad et al., 2014; Seol et al., 2017); thus, their migration and recruitment to the site of injury using these techniques would be greatly beneficial. Since the stiffest part of these cells is their nucleus, nuclear softening is also a promising approach to improve migration through the dense connective networks of the meniscus (Heo et al., 2020). Rather than improving cell recruitment by altering the cells, the matrix around the cell could also be loosened *via* local digestion (Qu et al., 2013, 2015). Similar techniques have been employed in cartilage repair (van de Breevaart Bravenboer et al., 2004; Seol et al., 2014; Liebesny et al., 2019), showing that loosening the network can not only enhance migration, but also improve the ability of the two torn edges to merge back together. This latter concept may be most important, as the dense meniscal network experiences little to no remodeling (Våben et al., 2020), whereas slightly degraded matrix can be remodeled more readily to integrate the two edges.

## Other Joint Factors That Influence Healing

Perhaps the most obvious environmental factor that has been implicated in meniscal healing potential is inflammation. The release of pro-inflammatory cytokines (e.g., interleukin-1, tumor necrosis factor-alpha) following soft tissue injury in the joint is well-documented (Irie et al., 2003; Haslauer et al., 2014; Ogura et al., 2016), both by the synovial/synovium cells and the injured tissue itself. In the meniscus specifically, integrative repair of the meniscus, both *in vitro* and *in vivo*, is reduced under inflamed environments (Hennerbichler et al., 2007; Riera et al., 2011), perhaps due to reduced proliferation and migration, and reduced capacity for meniscus specific matrix deposition and remodeling. Thus, intra-articular augmentation with, and perhaps even localized delivery of, anti-inflammatory agents may present promising improvements in repair success. Novel methods to deliver these drugs include capsules, carriers, spheres, both at the micro-scale and nano-scale to enhance retention, duration, and activity of both anti-inflammatory and pro-regenerative cues (Patel et al., 2019). Since both inflammation post-injury and the reparative process occur on the order of weeks to months, these prolonging attributes are especially helpful. Delaying meniscal repair procedures after injury, similar to what is done with anterior cruciate ligament reconstruction (Inoue et al., 2016), may help to delay repair until inflammation has subsided, improving the integrative nature, and thus long-term stability, of the repair.

Beyond the biological milieu within the joint, there are a variety of biomechanical joint factors at play. First and foremost, concomitant injuries, especially anterior cruciate ligament injuries and reconstruction, place a large mechanical burden on the meniscus (Dargel et al., 2007; Chen et al., 2017), and restoration of these tissues and their function are paramount to meniscal function and its ability to be repaired. This may also include an open-wedge osteotomy to correct varus or valgus malalignment (Jing et al., 2019; Rocha de Faria et al., 2021), to alleviate loads that are placed on one compartment of the knee. Furthermore, along the same lines, the rehabilitation timeline needs to be precisely controlled (Cavanaugh and Killian, 2012; Spang Iii et al., 2018); early overloading may cause re-tear before adequate tissue has been deposited to bridge the tear. Alternatively, since mechanical loading is beneficial to meniscal cell activity and matrix deposition (McNulty et al., 2010; Puetzer et al., 2012), a protocol that is too conservative can prohibit the increased regenerative capacity provided by loading. Timing prior to the procedure is also an important biomechanical consideration. While waiting can calm inflammation to enhance repair potential, it must be balanced with the increased risk of other injuries that can occur in this timeframe (Fok and Yau, 2013; Kolin et al., 2021; Prodromidis et al., 2021).

## Conclusion

The meniscal repair field has long cited the location along the radial axis, indicative of vascular content, as the sole determinant of treatment modality. I believe that other factors (circumferential disruption, dense matrix obstruction, and joint factors) may be just as influential to repair potential. Thus, the field would greatly benefit from additional clinical studies and reviews to better track outcomes with regards to these variables, which is fully possible with the increasing performance of inner zone repairs. Additional preclinical work, both *in vitro* and *in vivo*, will help to elucidate the healing potential of various tear configurations, especially as they relate to the alignment of collagen bundles, the relative density of the matrix, and the environmental inflammatory state. The recent shift to “save the meniscus” with repair techniques would greatly benefit from consideration of these alternative impediments to healing.
